# Daily physical activity associated with suicidal ideation in college students: an ecological momentary assessment study

**DOI:** 10.3389/fpsyg.2026.1852256

**Published:** 2026-05-29

**Authors:** Yimeng Ma, Wang Liao, Shuqing Ma, Laibing Lu, Haixia Wang

**Affiliations:** 1 University Student Mental Health Education Guidance Center, Henan Medical University, Xinxiang, China; 2 Innovation and Entrepreneurship College, Henan Medical University, Xinxiang, China; 3 College of Physical Education, Handan University, Handan, China; 4 Department of Sports Training, Henan Sport University, Zhengzhou, China

**Keywords:** childhood maltreatment, college students, ecological momentary assessment, physical activity, suicidal ideation, team-based activity

## Abstract

**Background:**

Suicidal ideation is common among college students and often fluctuates from day to day. Identifying short-term modifiable correlates is therefore important. Physical activity may be one such correlate, but its day-level association with suicidal ideation, the role of activity type, and the influence of childhood maltreatment remain unclear.

**Methods:**

This study used a two-stage design, including a baseline survey and a 14-day ecological momentary assessment (EMA) phase. Undergraduate students completed baseline measures of childhood maltreatment and suicidal ideation. Students with elevated suicidal ideation (BSI > = 6) who passed safety screening entered the EMA phase. During the 14-day period, suicidal ideation was assessed three times daily. Evening surveys assessed whether participants had engaged in at least 20 min of physical activity that day and whether the activity was individual or team-based. Multilevel mixed-effects models were used to examine day-level associations and the moderating role of childhood maltreatment.

**Results:**

Of 1,500 students who completed the baseline survey, 312 met the screening criterion for elevated suicidal ideation, 245 entered the EMA phase, and 208 were included in the final analyses. Daily physical activity was statistically associated with lower same-day evening suicidal ideation at the within-person level. When activity type was examined, both individual and team-based physical activity showed negative associations with evening suicidal ideation. Evidence for moderation by childhood maltreatment was strongest in the activity-type model. The overall physical activity × childhood maltreatment interaction reached only the conventional threshold, whereas the team-based physical activity × childhood maltreatment interaction was statistically significant; the inverse association between team-based activity and evening suicidal ideation became stronger as childhood maltreatment increased.

**Limitations:**

The study was conducted in a single university sample of Chinese college students with elevated suicidal ideation and relied on self-reported daily physical activity and a single-item EMA measure of suicidal ideation.

**Conclusion:**

Daily physical activity may be a relevant short-term behavioral correlate of suicidal ideation in college students with elevated suicidal ideation. Team-based activity may be particularly relevant for students with greater childhood maltreatment exposure.

## 1 Introduction

Suicidal ideation (SI) among university students is a major public health concern, particularly during this developmentally sensitive stage. The transition to college involves shifts in social roles, increased academic demands, and heightened uncertainty about the future, all of which may increase psychological vulnerability during emerging adulthood. Within this context, SI remains prevalent in university populations, including recent samples of Chinese college students (Mortier et al., 2018; Yao et al., 2023; Wu et al., 2021). Clinically, SI is especially critical as the most proximal cognitive precursor to suicidal behavior, making the identification of short-term correlates and modifiable intervention targets a central goal of suicide prevention research (Turecki and Brent, 2016).

Among distal risk factors, childhood maltreatment has been consistently identified as a major predictor of suicide risk. Experiences of abuse and neglect are associated with elevated risk of SI and related outcomes across the lifespan (Angelakis et al., 2019). This association may be particularly salient in university students, for whom college often represents the first period of managing the psychological consequences of early adversity outside the family context, frequently under ongoing academic stress and unstable social support. Recent studies in Chinese samples continue to show strong links between maltreatment and SI, partly explained by negative self-evaluation, depressive symptoms, and broader emotional distress (Liu et al., 2025; Pan et al., 2024). These findings highlight students with maltreatment histories as a high-risk subgroup and underscore the importance of identifying proximal, modifiable behavioral correlates.

A key limitation of the literature is methodological. Much of the research relies on retrospective and cross-sectional designs that implicitly treat SI as stable over time, an assumption that has increasingly been challenged. EMA studies show that SI can fluctuate substantially within hours, shaped by rapidly changing affective, cognitive, and interpersonal states (Kleiman et al., 2017; Portillo-Van Diest et al., 2025). Reviews further indicate that EMA reduces recall bias, improves ecological validity, and captures short-term dynamics missed by global reports (Winstone et al., 2024). For university students, whose distress is often episodic and context dependent, this is not only a methodological issue but also a conceptual one. If SI is dynamic, clinically relevant correlates may operate in real time at the within-person level rather than as stable between-person differences. Recent Frontiers work has likewise underscored the utility and feasibility of EMA for studying short-term suicidal ideation processes in real-world contexts (Ernst et al., 2022).

Physical activity is a plausible candidate for such a proximal behavioral correlate. It is low-cost, scalable, and generally associated with better mental health. However, its association with SI remains mixed, partly because most studies conceptualize activity as a stable lifestyle trait rather than a time-varying daily exposure (Fabiano et al., 2023; Jacob et al., 2024). This distinction is critical, as daily activity may be more relevant to momentary SI than average long-term levels, yet such effects are largely inaccessible to retrospective designs. Moreover, physical activity differs socially: individual and team-based activities may share physiological features but diverge in interpersonal processes. Recent Frontiers in Psychology studies in Chinese student samples similarly report inverse associations between physical exercise and suicidal ideation and point to potential roles for emotion regulation, basic psychological needs, and meaning in life (Ou et al., 2025; Zhang et al., 2025).

This distinction aligns with interpersonal theories of suicide. Team-based activities provide opportunities for coordination, shared goals, and low-intensity social interaction, and are associated with broader psychosocial outcomes beyond physical movement (Eather et al., 2023). For individuals with childhood maltreatment, these features may be especially relevant, given disruptions in attachment, trust, perceived support, and sensitivity to social threat (Angelakis et al., 2019; Liu et al., 2025; Pan et al., 2024). Team sport may offer a structured context for experiencing belonging without intensive self-disclosure, whereas individual activity may primarily relate to mood regulation. However, EMA-based evidence examining whether short-term associations between daily physical activity and SI differ by activity type, and whether such associations vary as a function of maltreatment exposure, remains limited. This emphasis on the social context of sport is also consistent with recent Frontiers evidence suggesting that sports participation and especially team-based competitive activities may foster belonging and are associated with suicide-related or self-harm-related outcomes (Huo et al., 2024; Liang et al., 2026).

The present study used a 14-day EMA design to examine associations between daily physical activity and same-day suicidal ideation, whether team-based activity shows stronger associations than individual activity, and whether these associations are moderated by childhood maltreatment. By focusing on within-person dynamics in naturalistic settings, this study aims to move beyond static models of suicide risk and identify a potential, scalable target for real-time intervention.

### 1.1 Hypotheses

First, we hypothesized that same-day evening SI would be lower on days with physical activity than on inactive days. Second, we hypothesized that team-based activity would show stronger negative associations with same-day suicidal ideation than individual activity. Third, we hypothesized that childhood maltreatment would moderate these associations, such that inverse associations, especially for team-based activity, would be stronger among students with higher maltreatment exposure.

## 2 Methods

### 2.1 Participants and procedure

The study employed a two-stage design consisting of a baseline screening phase followed by a 14-day ecological momentary assessment (EMA) phase. Participants were recruited from a university in Xinxiang, China, through campus online platforms and mental health education courses. Data collection was conducted from March to May 2025. An initial online baseline survey was administered to 1,500 undergraduate students to assess demographic characteristics, childhood maltreatment, and baseline suicidal ideation.

A total of 312 students scored 6 or higher on the Beck Scale for Suicide Ideation (BSI), indicating elevated current suicidal ideation, and proceeded to eligibility screening for the EMA phase. Given the sensitivity of the study, a structured safety screening procedure was conducted prior to EMA enrollment. Participants were excluded if they reported (a) an active suicide plan, (b) a suicide attempt within the past 6 months, or (c) severe substance misuse or acute psychotic symptoms. Individuals meeting these criteria were withdrawn from the study and referred for professional support through the university’s Psychological Counseling Center and its affiliated psychiatric hospital.

Following screening, 245 eligible students were invited to participate in the 14-day EMA phase. Prior to enrollment, participants were informed about the study procedures, data confidentiality, and their right to withdraw at any time without penalty, and provided written informed consent. EMA compliance was evaluated after the monitoring period, and participants with completion rates below 60% were excluded from the final analytic sample, consistent with common practice in intensive longitudinal research.

During the 14-day EMA monitoring phase, participant safety was managed using a predefined risk-management procedure. Participants were provided with contact information for the university Psychological Counseling Center and affiliated psychiatric hospital. EMA responses were reviewed by trained research staff, and any report approaching the upper end of the 0–100 suicidal ideation scale or any indication of acute risk would trigger immediate escalation to the study supervisor and the university mental health service for contact, risk assessment, and referral.

The final sample included 208 students (retention rate = 84.9%). The mean age was 20.52 years (SD = 1.42), with 113 females (54.3%) and 95 males (45.7%). Across the 14-day period, 2,912 day-level observations were expected, of which 2,358 were completed (completion rate = 81.0%). The study protocol was approved by the Institutional Review Board of Xinxiang Medical University, now renamed Henan Medical University (Approval No. XYLL-20230533).

### 2.2 Measures

#### 2.2.1 Baseline assessments

Childhood maltreatment before age 18 was assessed using the Childhood Trauma Questionnaire-Short Form (CTQ-SF; Bernstein et al., 2003). The CTQ-SF is a 28-item self-report measure covering five domains: emotional abuse, physical abuse, sexual abuse, emotional neglect, and physical neglect. Items are rated on a 5-point Likert scale (1 = never true to 5 = very often true), with higher scores indicating greater maltreatment severity. The Chinese version has demonstrated acceptable reliability and validity (He et al., 2019). In the present study, Cronbach’s alpha for the total scale was 0.89.

Baseline suicidal ideation was assessed using the Beck Scale for Suicide Ideation (BSI; Beck et al., 1979), a 19-item measure evaluating suicidal thoughts over the past week. Items are scored on a 3-point scale (0–2), with higher scores indicating greater severity. A cutoff score of 6 or higher was used to define elevated suicidal ideation, consistent with prior Chinese research (Li et al., 2011). Cronbach’s alpha in the present sample was 0.86.

#### 2.2.2 Ecological momentary assessment variables

During the EMA phase, suicidal ideation was assessed three times per day using a single-item prompt: “To what extent have you had thoughts of killing yourself since the last prompt?” Responses were recorded on a visual analogue scale ranging from 0 (not at all) to 100 (very much). Single-item EMA measures are widely used in intensive longitudinal suicide research to reduce participant burden while maintaining sensitivity to short-term within-person fluctuations (Kleiman and Nock, 2018; Kleiman et al., 2017).

Daily physical activity was assessed in the evening EMA survey. A 20-min threshold was used to define an active day, and this threshold was treated as an exploratory cut-off in the present study. To evaluate whether the findings depended on this operationalization, sensitivity analyses were conducted using alternative thresholds of ≥10, ≥30, and ≥60 min. On days when participants reported physical activity, they further classified the activity as either individual (e.g., running or weight training) or team-based (e.g., basketball or soccer). The survey did not include a separate “both” option; participants selected the activity type that best characterized their main activity that day. Activity type was therefore analyzed as a single daily category to distinguish solitary and socially embedded forms of activity in subsequent analyses. The survey recorded whether participants engaged in physical activity and the total duration of activity on that day, but it did not record the exact time of day or objective intensity of the activity. Therefore, the present analyses could not distinguish morning from afternoon/evening physical activity or examine intensity-specific associations.

### 2.3 Data analysis strategy

Because the EMA data had a hierarchical structure, with repeated day-level observations nested within individuals, the analyses were conducted using multilevel mixed-effects models (Gueorguieva and Krystal, 2004; Myin-Germeys et al., 2018). An unconditional null model was first estimated for evening suicidal ideation to calculate the intraclass correlation coefficient (ICC) and confirm the appropriateness of multilevel modeling.

The primary analyses were conducted in a stepwise manner. First, a main-effects model was estimated to examine whether overall daily physical activity predicted same-day evening suicidal ideation. Second, a model simultaneously including individual physical activity and team-based physical activity was fitted to compare their unique within-person associations with evening suicidal ideation. Third, a cross-level interaction model was estimated to test whether childhood maltreatment moderated the within-person association between physical activity and evening suicidal ideation. Significant interactions were further probed using simple slopes analyses and Johnson-Neyman analysis (Bauer and Curran, 2005).

To distinguish within-person from between-person effects, day-level physical activity variables were person-mean centered, and the corresponding person-mean terms were entered simultaneously in the models (Enders and Tofighi, 2007; Wang and Maxwell, 2015). Childhood maltreatment was grand-mean centered. All adjusted models controlled for lagged evening suicidal ideation, study day, age, gender, and baseline suicidal ideation. Missing EMA observations were not imputed; analyses were based on all available completed day-level records with non-missing evening suicidal ideation, and models including lagged evening suicidal ideation additionally required non-missing lagged values. Participants with EMA completion rates below 60% were excluded. All reported models included random intercepts for participants, and random slopes for the within-person physical activity effect were tested but not retained. Models were estimated using maximum likelihood, and statistical significance was set at *p* < 0.05 (two-tailed). Sensitivity analyses were conducted using alternative physical activity thresholds of ≥10, ≥30, and ≥60 min. Standardized effect sizes and model diagnostics are reported in the Supplementary Materials.

## 3 Results

### 3.1 Participant flow and descriptive statistics

A total of 1,500 students completed the baseline survey, of whom 312 met the inclusion criterion (BSI > = 6) and entered the eligibility screening stage. After safety screening, 245 students entered the EMA phase. Following the 14-day monitoring period, 37 participants were excluded because their compliance rate fell below the predefined 60% threshold, resulting in a final analytic sample of 208 participants. The sample included 113 females (54.3%) and 95 males (45.7%), with a mean age of 20.52 years (SD = 1.42). The mean CTQ total score was 42.76 (SD = 9.62), and the mean baseline BSI score was 8.56 (SD = 1.68; range = 6–13). Descriptive characteristics of the analytic sample are presented in Table 1.

**Table 1:** Descriptive characteristics of the analytic sample, EMA compliance, and EMA/activity distributions.

Characteristic	Statistic	Value
Sample characteristics		
Baseline survey participants	*n*	1,500
Met BSI criterion (≥ 6)	*n*	312
Entered EMA phase	*n*	245
Final analytic sample	*n*	208
Female	*n* (%)	113 (54.3)
Male	*n* (%)	95 (45.7)
Age	Mean (SD)	20.52 (1.42)
CTQ total score	Mean (SD)	42.76 (9.62)
Baseline BSI score	Mean (SD)	8.56 (1.68)
Baseline BSI score	Range	6–13
EMA compliance and observations		
Scheduled day-level records	*n*	2,912
Completed day-level records	*n*	2,358
Overall completion rate	%	81.0
Non-missing momentary SI ratings	*n*	6,495
EMA suicidal ideation		
EMA SI ratings	Mean (SD)	13.44 (10.16)
EMA SI ratings	Range	0.00–56.24
EMA SI ratings	Mean within-person variance	43.27
Daily physical activity		
Active study days	Mean % (SD)	34.9 (18.6)
Team-based PA among active days	%	41.4
Individual PA among active days	%	58.6
Activity duration on active days	*n*	821
Activity duration on active days	Mean (SD)	42.44 (16.32)
Activity duration on active days	Range (min–max)	20–91

CTQ, Childhood Trauma Questionnaire; BSI, Beck Scale for Suicide Ideation; EMA, ecological momentary assessment; SI, suicidal ideation; PA, physical activity. Percentages for team-based and individual physical activity were calculated among active completed days only. EMA suicidal ideation statistics were calculated across all non-missing morning, afternoon, and evening ratings (*n* = 6,495). Mean within-person variance was calculated as the average person-specific variance across all non-missing EMA suicidal ideation ratings.

To evaluate potential compliance-related selection bias, participants retained in the final analytic sample were compared with those excluded because of EMA compliance below 60% on baseline CTQ total scores and baseline BSI scores. No significant differences were observed between retained and excluded participants in CTQ total scores or baseline BSI scores, and effect sizes were small (Supplementary Table S4), suggesting no strong evidence of baseline differences in childhood maltreatment or suicidal ideation severity.

Across the 14-day EMA period, 2,358 of 2,912 scheduled day-level records were completed, corresponding to a day-level completion rate of 81.0%. A total of 6,495 non-missing momentary suicidal ideation ratings were available. Participants reported physical activity on 34.9% of study days (SD = 18.6%). Among active days, 41.4% involved team-based physical activity and 58.6% involved individual physical activity.

Across the EMA phase, the 6,495 non-missing suicidal ideation ratings had a mean of 13.44 (SD = 10.16), with a range of 0.00 to 56.24. Exact zero values accounted for 738 ratings (11.4%), and the mean within-person variance of EMA suicidal ideation was 43.27, indicating substantial short-term fluctuation across repeated assessments. On active days (n = 821), physical activity duration averaged 42.44 min (SD = 16.32; range = 20–91). Histograms of baseline BSI total scores, EMA suicidal ideation ratings, and activity duration on active days are provided in Supplementary Figures S4–S6.

### 3.2 Null model

An unconditional null model was first estimated for evening suicidal ideation. As shown in Table 2, the random intercept variance was 61.479 and the residual variance was 31.269, yielding an intraclass correlation coefficient (ICC) of 0.663. Thus, 66.3% of the variance in evening suicidal ideation was attributable to between-person differences, whereas 33.7% reflected within-person day-to-day fluctuations. These results supported the use of multilevel modeling for the subsequent analyses.

**Table 2:** Null-model summary for evening suicidal ideation.

Parameter	Estimate
Random intercept variance	61.479
Residual variance	31.269
Intraclass correlation coefficient (ICC)	0.663
Between-person variance (%)	66.3
Within-person variance (%)	33.7

### 3.3 Main effects of daily physical activity on evening suicidal ideation

To examine whether daily physical activity was associated with same-day evening suicidal ideation, multilevel mixed-effects models were estimated using 2,131 day-level observations nested within 208 participants. Missing EMA observations were not imputed; analyses were based on all available completed day-level records with non-missing evening suicidal ideation and lagged evening suicidal ideation. All reported models included participant-level random intercepts, and random-slope specifications for the within-person physical activity effect were examined but not retained. All models adjusted for lagged evening suicidal ideation, study day, age, gender, and baseline suicidal ideation.

As shown in Table 3, in the model including overall daily physical activity, the within-person effect of physical activity was significant and negative (*B* = −1.780, SE = 0.268, *z* = −6.643, *p* < 0.001, 95% CI [−2.305, −1.255]), whereas the between-person effect of person-mean physical activity was not significant (*B* = −2.629, SE = 1.609, *z* = −1.634, *p* = 0.102, 95% CI [−5.782, 0.525]). Lagged evening suicidal ideation (*B* = 0.413, SE = 0.025, *z* = 16.236, *p* < 0.001, 95% CI [0.363, 0.463]) and baseline suicidal ideation (*B* = 1.212, SE = 0.190, *z* = 6.372, *p* < 0.001, 95% CI [0.839, 1.585]) were significant positive covariates, whereas study day, age, and gender were not significant.

**Table 3:** Multilevel mixed-effects models predicting evening suicidal ideation from daily physical activity.

Predictor	*B*	*SE*	*z*	*p*	95% CI Lower	95% CI Upper
Model 1. Overall daily physical activity						
Intercept	−2.503	4.686	−0.534	0.593	−11.687	6.681
Lagged evening suicidal ideation	0.413	0.025	16.236	< 0.001	0.363	0.463
Study day	−0.025	0.029	−0.863	0.388	−0.083	0.032
Age	0.046	0.216	0.211	0.833	−0.378	0.469
Gender (female = 1)	−0.341	0.615	−0.555	0.579	−1.546	0.864
Baseline suicidal ideation	1.212	0.190	6.372	< 0.001	0.839	1.585
Within-person daily physical activity	−1.780	0.268	−6.643	< 0.001	−2.305	−1.255
Person-mean daily physical activity	−2.629	1.609	−1.634	0.102	−5.782	0.525
Model 2. Individual and team-based physical activity						
Intercept	−1.573	4.688	−0.335	0.737	−10.762	7.616
Lagged evening suicidal ideation	0.414	0.025	16.246	< 0.001	0.364	0.464
Study day	−0.027	0.029	−0.903	0.366	−0.084	0.031
Age	0.011	0.216	0.051	0.959	−0.412	0.434
Gender (female = 1)	−0.389	0.611	−0.637	0.524	−1.587	0.809
Baseline suicidal ideation	1.183	0.190	6.240	< 0.001	0.811	1.555
Within-person individual physical activity	−1.577	0.321	−4.912	< 0.001	−2.206	−0.948
Within-person team-based physical activity	−2.061	0.362	−5.695	< 0.001	−2.770	−1.351
Person-mean individual physical activity	−4.856	2.144	−2.266	0.023	−9.058	−0.655
Person-mean team-based physical activity	0.848	2.734	0.310	0.756	−4.511	6.208

When individual and team-based physical activity were entered simultaneously, both showed significant negative within-person associations with evening suicidal ideation. Individual physical activity was associated with lower evening suicidal ideation (*B* = −1.577, SE = 0.321, *z* = −4.912, *p* < 0.001, 95% CI [−2.206, −0.948]), as was team-based physical activity (*B* = −2.061, SE = 0.362, *z* = −5.695, *p* < 0.001, 95% CI [−2.770, −1.351]). At the between-person level, person-mean individual physical activity was significant (*B* = −4.856, SE = 2.144, *z* = −2.266, *p* = 0.023, 95% CI [−9.058, −0.655]), whereas person-mean team-based physical activity was not (*B* = 0.848, SE = 2.734, *z* = 0.310, *p* = 0.756, 95% CI [−4.511, 6.208]). Lagged evening suicidal ideation (*B* = 0.414, SE = 0.025, *z* = 16.246, *p* < 0.001, 95% CI [0.364, 0.464]) and baseline suicidal ideation (*B* = 1.183, SE = 0.190, *z* = 6.240, *p* < 0.001, 95% CI [0.811, 1.555]) remained significant positive covariates, whereas study day, age, and gender were not significant.

Model fit was similar for the overall physical activity model (AIC = 13651.306, BIC = 13707.950) and the activity-type model (AIC = 13651.545, BIC = 13719.517). These findings support Hypothesis 1 and provide partial support for Hypothesis 2.

Sensitivity analyses were conducted using alternative activity-duration thresholds of ≥10, ≥30, and ≥60 min. Re-estimating the primary mixed-effects model yielded a consistent pattern across thresholds, with active days remaining associated with lower same-day evening suicidal ideation. Specifically, the coefficient for active days was *β* = −1.780 (SE = 0.268, 95% CI [−2.305, −1.255], *p* < 0.001) for both the ≥10-min and ≥20-min thresholds, β = −1.352 (SE = 0.292, 95% CI [−1.924, −0.780], *p* < 0.001) for the ≥30-min threshold, and β = −1.275 (SE = 0.558, 95% CI [−2.369, −0.181], *p* = 0.022) for the ≥60-min threshold (Table 4). The identical estimates for the ≥10-min and ≥20-min thresholds reflect the absence of daily observations with activity duration between 1 and 19 min in the analytic sample. In addition, no daily observations in the present dataset were coded as involving both individual and team-based activity. Additional information on missing-data handling, random-effects comparisons, standardized effect sizes, and model diagnostics is provided in Supplementary Tables S1–S3 and Supplementary Figures S1–S3.

**Table 4:** Sensitivity analyses of alternative physical activity cut-offs in the main mixed-effects model.

Threshold	Active days, n	Inactive days, n	Beta	SE	95% CI	*p*
≥10	741	1,390	−1.780	0.268	[−2.305, −1.255]	< 0.001
≥20	741	1,390	−1.780	0.268	[−2.305, −1.255]	< 0.001
≥30	551	1,580	−1.352	0.292	[−1.924, −0.780]	< 0.001
≥60	116	2015	−1.275	0.558	[−2.369, −0.181]	0.022

Linear mixed-effects models with random intercepts for participants were estimated using completed daily records with non-missing evening SI (2,131 days from 208 participants). Covariates were lagged evening SI, study day, age, gender, and baseline BSI score. Negative beta values indicate lower evening suicidal ideation on active days. The results for the ≥10 and ≥20 min thresholds were identical because no daily observations in the analytic sample fell between 1 and 19 min of physical activity.

### 3.4 Moderating effect of childhood maltreatment on the association between daily physical activity and evening suicidal ideation

A cross-level interaction model was estimated to test whether childhood maltreatment moderated the within-person association between daily physical activity and evening suicidal ideation. As shown in Table 5, childhood maltreatment was positively associated with evening suicidal ideation at the between-person level (*B* = 0.182, SE = 0.037, *z* = 4.809, *p* < 0.001, 95% CI [0.109, 0.258]). The interaction between within-person daily physical activity and childhood maltreatment was negative and reached the conventional threshold for statistical significance (*B* = −0.053, SE = 0.027, *z* = −1.960, *p* = 0.050, 95% CI [−0.107, 0.000]).

**Table 5:** Multilevel model testing the moderating effect of childhood maltreatment on the association between daily physical activity and evening suicidal ideation.

Predictor	*B*	*SE*	*z*	*p*	95% CI lower	95% CI upper
Intercept	2.873	4.566	0.629	0.529	−6.076	11.822
Lagged evening SI	0.413	0.025	16.313	< 0.001	0.364	0.463
Study day	−0.028	0.029	−0.962	0.336	−0.086	0.029
Age	0.035	0.205	0.173	0.863	−0.366	0.436
Gender (female = 1)	−0.649	0.586	−1.107	0.268	−1.797	0.499
Baseline BSI	0.628	0.211	2.972	0.003	0.214	1.042
Daily PA (within-person)	−1.777	0.268	−6.637	< 0.001	−2.301	−1.252
Daily PA (person-mean)	−2.590	1.522	−1.701	0.089	−5.574	0.394
Childhood maltreatment	0.182	0.037	4.874	< 0.001	0.109	0.255
Daily PA × childhood maltreatment	−0.053	0.027	−1.960	0.050	−0.107	0.000

The main within-person effect of daily physical activity remained significant after inclusion of the interaction term. Model fit improved relative to the main-effects model (AIC = 13628.112, BIC = 13696.084; likelihood-ratio test: chi-square(2) = 27.194, *p* < 0.001). Although the interaction reached the conventional threshold for significance, this finding should be interpreted cautiously.

### 3.5 Differential moderation by individual versus team-based physical activity

To examine whether the moderating effect of childhood maltreatment differed by activity type, an additional multilevel model simultaneously included individual physical activity, team-based physical activity, childhood maltreatment, and the corresponding interaction terms. As shown in Table 6, the interaction between individual physical activity and childhood maltreatment was not significant (*B* = 0.019, SE = 0.032, *z* = 0.604, *p* = 0.546, 95% CI [−0.043, 0.082]). In contrast, the interaction between team-based physical activity and childhood maltreatment was significant and negative (*B* = −0.156, SE = 0.037, *z* = −4.213, *p* < 0.001, 95% CI [−0.228, −0.083]).

**Table 6:** Differential moderation by individual versus team-based physical activity.

Predictor	*B*	*SE*	*z*	*p*	95% CI lower	95% CI upper
Individual PA × childhood maltreatment	0.019	0.032	0.604	0.546	−0.043	0.082
Team-based PA × childhood maltreatment	−0.156	0.037	−4.213	< 0.001	−0.228	−0.083

Model fit improved relative to the corresponding main-effects model (AIC = 13615.157, BIC = 13700.123; likelihood-ratio test: chi-square(3) = 42.388, *p* < 0.001). A direct comparison of the two interaction terms indicated that the moderation effect was significantly stronger for team-based than for individual physical activity (Wald chi-square(1) = 17.331, *p* < 0.001). Subsequent probing analyses therefore focused on team-based physical activity.

### 3.6 Probing the significant interaction: simple slopes and Johnson-Neyman analyses

The significant interaction between team-based physical activity and childhood maltreatment was further examined using simple slopes and Johnson-Neyman analyses. As shown in Table 7, at low levels of childhood maltreatment, the within-person association between team-based physical activity and evening suicidal ideation was not significant (*B* = −0.415, SE = 0.528, *z* = −0.787, *p* = 0.431, 95% CI [−1.450, 0.619]). At high levels of childhood maltreatment, the association was significant and negative (*B* = −3.419, SE = 0.485, *z* = −7.046, *p* < 0.001, 95% CI [−4.371, −2.468]).

**Table 7:** Simple slopes for the interaction between team-based physical activity and childhood maltreatment predicting evening suicidal ideation.

Condition	CTQ value	*B*	*SE*	*z*	*p*	95% CI lower	95% CI upper
Low childhood maltreatment (−1 SD)	33.14	−0.415	0.528	−0.787	0.431	−1.450	0.619
High childhood maltreatment (+1 SD)	52.38	−3.419	0.485	−7.046	< 0.001	−4.371	−2.468

**Figure 1:**
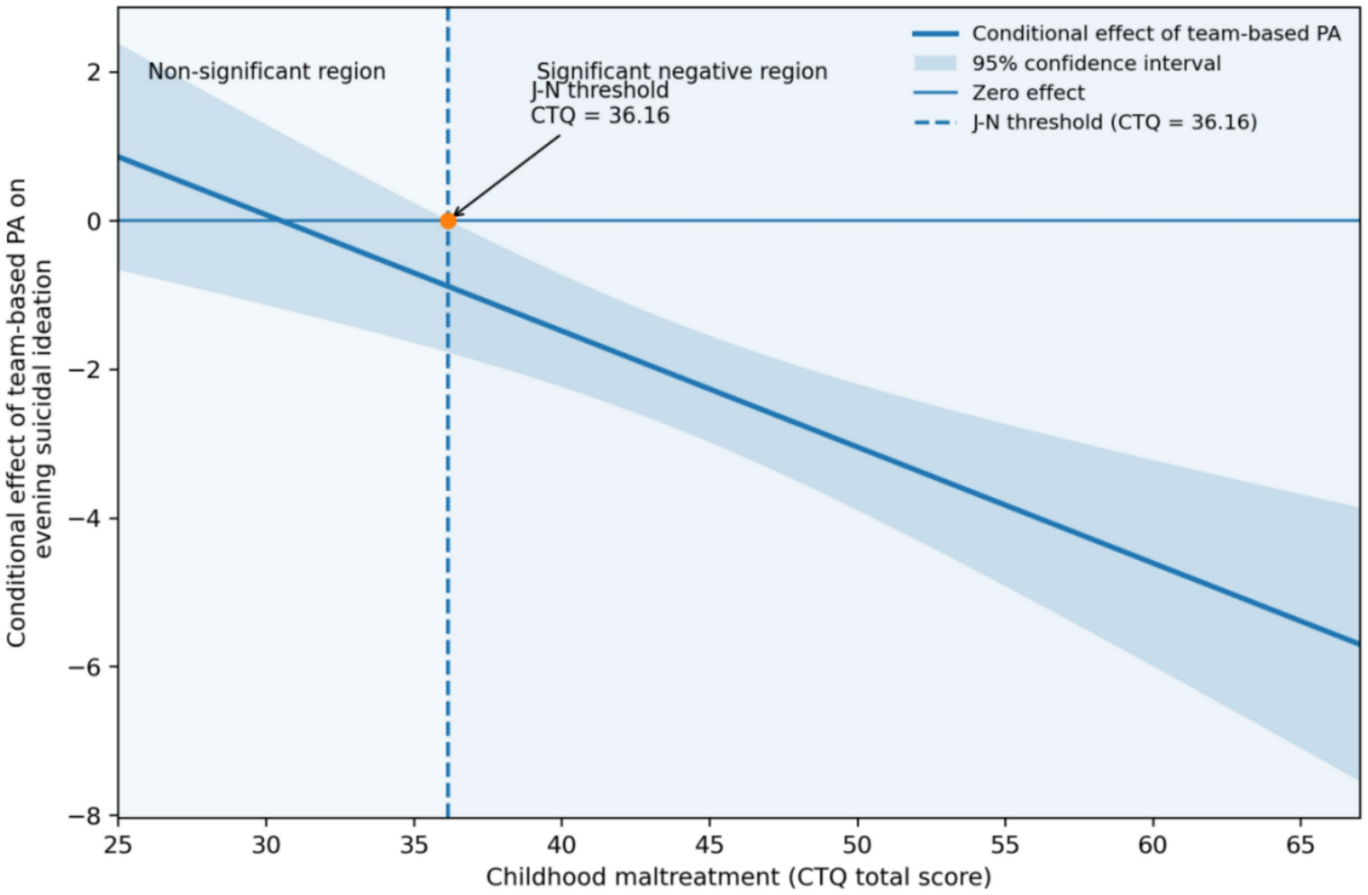
Johnson–Neyman plot of the conditional effect of team-based physical activity on evening suicidal ideation across levels of childhood maltreatment. Shaded area represents the 95% confidence interval. The dashed vertical line indicates the Johnson–Neyman threshold (CTQ = 36.16).

Johnson-Neyman analysis showed that the association became significantly negative when childhood maltreatment exceeded a CTQ score of 36.16 within the observed range of 25 to 67. The conditional effect is shown in Figure 1.

## 4 Discussion

### 4.1 Association between physical activity and suicidal ideation

The findings of this study indicate that daily physical activity was associated with lower same-day evening suicidal ideation in college students. At the within-person level, evening suicidal ideation was lower on days when participants engaged in physical activity than on days when they did not, even after adjustment for prior-evening suicidal ideation and baseline suicidal ideation. This pattern fits well with work showing that suicidal ideation is not a fixed, trait-like phenomenon, but a fluctuating state that can shift meaningfully across hours and days. EMA studies have therefore emphasized the value of real-time designs for identifying near-term risk and factors associated with lower suicidal ideation that are difficult to capture with retrospective methods (Kleiman and Nock, 2018; Kleiman et al., 2017).

Against this background, the present study extends the literature in an important way. Prior research has linked physical activity to lower suicide-related risk, and recent evidence syntheses suggest that exercise interventions are associated with improved suicide-related outcomes, although findings for suicidal ideation remain mixed, particularly when physical activity is treated as a relatively stable characteristic rather than a time-varying behavior (Fabiano et al., 2023, 2024; Vancampfort et al., 2018). The current results suggest that the association between daily physical activity and same-day suicidal ideation may be more apparent at the day level. In other words, the relevant question may not only be whether more active students are less suicidal on average, but also whether being active today is associated with lower suicidal ideation tonight. This interpretation is also in line with recent Frontiers studies showing negative associations between physical exercise and suicidal ideation in Chinese college and medical student samples (Ou et al., 2025; Zhang et al., 2025).

This interpretation also underscores the importance of temporal scale. If suicidal ideation unfolds in rapid and context-sensitive fluctuations, clinically relevant day-level correlates may need to be conceptualized at a comparable temporal resolution (Kleiman and Nock, 2018; Kleiman et al., 2017). Daily physical activity may be relevant not simply because more active students are generally healthier, but because activity on a given day may be associated with affective arousal, attention, behavioral activation, and stress regulation in ways that matter for suicidal ideation. Although these mechanisms were not tested directly, this interpretation is consistent with evidence that physical activity engagement is associated with lower stress-related affective responses and emotional recovery (Bernstein and McNally, 2018; Gerstberger et al., 2023).

Importantly, the present study assessed physical activity using a brief daily self-report based on duration and activity type, but did not objectively record exercise intensity. This measurement limitation should be considered when interpreting the observed associations. Activities with the same duration may differ substantially in physiological load, perceived exertion, and affective consequences, and these differences could influence their association with evening suicidal ideation. For example, higher-intensity activity may be more strongly related to physiological arousal and post-exercise affective change, whereas lower-intensity activity may primarily reflect behavioral activation or social engagement. Because intensity was not measured, the present study could not distinguish whether the observed associations were driven by duration, intensity, activity context, or their combination.

The non-significant between-person effect for overall physical activity is also informative. In this sample, the more clinically relevant signal appeared to lie in day-to-day variation rather than in average activity levels. For suicide prevention research, this distinction matters because it shifts attention from broad lifestyle differences to potentially actionable behaviors in everyday life. Among college students, whose distress is often episodic and context dependent, such a within-person perspective may be especially useful for identifying practical and scalable intervention targets. This interpretation is also compatible with broader work suggesting that sport and physical activity can confer psychological and social benefits beyond physical health alone (Andersen et al., 2019; Eather et al., 2023).

### 4.2 Moderating role of childhood maltreatment in the association between physical activity and suicidal ideation

The findings also suggest that childhood maltreatment may be relevant not only as a distal risk factor for suicidal ideation, but also as a vulnerability context that may shape the day-level association between physical activity and same-day suicidal ideation. However, the overall daily physical activity × childhood maltreatment interaction reached only the conventional threshold for statistical significance (*p* = 0.050; 95% CI upper bound = 0.000), and this result should therefore be interpreted cautiously. Rather than providing definitive evidence of moderation for overall daily physical activity, the finding suggests a possible pattern in which students with higher childhood maltreatment tended to show a stronger inverse association between daily physical activity and evening suicidal ideation. This interpretation is consistent with a substantial literature linking childhood maltreatment to elevated suicidal ideation and related suicidal outcomes across both youth and adulthood (Angelakis et al., 2020; Serebriakova et al., 2025).

One cautious interpretation is that daily physical activity may be especially relevant for students with maltreatment histories because it may provide a near-term regulatory resource in the context of greater developmental vulnerability. Childhood maltreatment has been associated with later difficulties in emotion regulation, coping, and psychosocial adjustment, whereas resilience-oriented research has highlighted adaptive coping, social support, and regulatory capacities as important resilience-related factors following adversity (Meng et al., 2018; Su et al., 2020). Within this framework, physical activity may be associated with lower suicidal ideation partly through stress-related and cognitive-emotional processes on a given day, particularly among those whose baseline vulnerability is higher. This interpretation is also in line with experience-sampling evidence showing that physical activity engagement is associated with lower affective responses to daily stress (Gerstberger et al., 2023). Nevertheless, because the overall interaction was marginal, this explanation should be regarded as provisional until replicated in future studies.

These results may also help explain why previous findings on physical activity and suicidal ideation have sometimes been inconsistent. Given the marginal nature of the overall interaction, childhood maltreatment should not be described as a definitive boundary condition for the association between overall daily physical activity and suicidal ideation. Instead, the present findings raise the possibility that adversity-related vulnerability may contribute to heterogeneity in day-level associations between physical activity and suicidal ideation. This possibility requires confirmation in future studies with larger samples, repeated assessments of potential mechanisms, and designs capable of clarifying temporal ordering.

### 4.3 Differential roles of team-based and individual physical activity in the context of childhood maltreatment

More robust evidence for moderation emerged when activity type was distinguished. Although both individual and team-based physical activity were associated with lower evening suicidal ideation at the within-person level, only the interaction between team-based activity and childhood maltreatment was statistically significant. Thus, the activity-type analyses suggest that the possible moderation by childhood maltreatment may be more evident for socially embedded forms of activity than for individual activity. This distinction is theoretically meaningful because team-based activities involve more than physical exertion alone; they typically include coordination, shared goals, repeated interaction, and opportunities for social connection. Reviews of sport participation have similarly suggested that team sport may provide psychological and social benefits that extend beyond the effects of movement itself (Andersen et al., 2019; Eather et al., 2023).

This pattern may be especially relevant for students with childhood maltreatment because early adversity often affects later interpersonal functioning, including trust, attachment security, perceived support, and sensitivity to rejection or threat. In this regard, the current findings are compatible with the interpersonal theory of suicide, which highlights thwarted belongingness as a central pathway to suicidal ideation (Ma et al., 2016; Van Orden et al., 2010). More broadly, social disconnection and isolation have been consistently linked to suicidal thoughts and behaviors (Calati et al., 2019). Team-based physical activity may therefore be associated with lower suicidal ideation not only because it involves behavioral activation, but also because it places activity within an immediately social context in which students may experience coordination, acceptance, and a sense of belonging. In parallel, recent Frontiers evidence suggests that competitive team sports are associated with self-harm-related outcomes partly through perceived belonging (Liang et al., 2026).

This interpretation may also help explain why individual physical activity, although associated with lower evening suicidal ideation, did not show the same pattern of strengthening under childhood maltreatment. Individual activity may still be associated with lower suicidal ideation through mechanisms such as distraction, physiological regulation, or emotion regulation, but it does not necessarily provide the same degree of interpersonal contact or belonging-related feedback. By contrast, team-based activity may combine self-regulatory features with socially embedded experiences that are especially relevant for students with developmental interpersonal vulnerabilities. Existing sport research is consistent with this view: team sport participation has been linked to lower depressive symptoms and better belonging-related outcomes, and the interpersonal dimensions of sport are increasingly recognized as key pathways to mental health associations (Bang et al., 2024; Bhargav and Swords, 2022).

Taken together, these findings suggest that suicide prevention research may benefit from distinguishing between physical activity as a form of energy expenditure and physical activity as a socially embedded daily experience. For college students with greater developmental adversity, activities that involve connection, cooperation, and recurring social engagement may offer more than solitary exercise alone. Although the present findings do not support strong causal conclusions, they underscore the relevance of the interpersonal context of physical activity for interventions aimed at reducing short-term suicidal ideation in vulnerable young adults. Because physical activity and evening suicidal ideation were measured concurrently, future studies using microrandomized trials or time-lagged designs are needed to clarify the temporal sequence of these associations.

## 5 Limitations and future directions

Several limitations should be noted. First, the sample was drawn from a single university and included only students with elevated suicidal ideation, which may limit generalizability. Second, the study was observational. Because physical activity and evening suicidal ideation were assessed concurrently, causal inference and temporal ordering cannot be established; future studies should use microrandomized trials or time-lagged designs. Relatedly, physical activity was recorded as a daily self-report without information on the exact timing of exercise, preventing lag-effect or time-of-day sensitivity analyses comparing morning, afternoon, or evening activity in relation to evening suicidal ideation. Third, depressive symptoms and antidepressant medication use were not assessed, although both may confound or partly explain associations between physical activity and suicidal ideation. Fourth, physical activity was operationalized using duration-based thresholds and mutually exclusive activity-type categories, which may have reduced information on activity amount and mixed activity patterns. Exercise intensity was also not objectively recorded, potentially introducing measurement error and obscuring intensity-specific associations. Future studies should incorporate time-stamped activity logs or wearable devices to capture activity timing, duration, and intensity more accurately. Fifth, suicidal ideation was assessed using a single EMA item, which may not fully capture the complexity of short-term suicidal ideation fluctuations.

## 6 Conclusion

Daily physical activity was associated with lower same-day evening suicidal ideation among college students with elevated suicidal ideation. Both individual and team-based physical activity were associated with lower same-day evening suicidal ideation. The overall physical activity × childhood maltreatment interaction reached the conventional threshold for significance and should be interpreted cautiously, whereas the activity-type analyses indicated that moderation by childhood maltreatment was more evident for team-based activity. In particular, the negative association between team-based physical activity and evening suicidal ideation became stronger as childhood maltreatment increased. These findings suggest that daily physical activity, especially team-based activity, may be a relevant short-term behavioral correlate of suicidal ideation among vulnerable college students.

## Data Availability

The original contributions presented in the study are included in the article/Supplementary material, further inquiries can be directed to the corresponding author.
