# Supplementary material for: Daily physical activity associated with suicidal ideation in college students: an ecological momentary assessment study
Source: Front Psychol. 2026 May 29;17:1852256. doi: 10.3389/fpsyg.2026.1852256 (PMC13260014; doi:10.3389/fpsyg.2026.1852256)
Supplement: Supplementary file 1 [file Supplementary_file_1.docx]

**Supplementary Materials**

This supplement provides additional information for the revised manuscript on missing-data handling, the random-effects structure of the multilevel models, standardized effect-size indices, model diagnostics, compliance-related comparisons between retained and excluded EMA participants, and distributional assessments of baseline BSI scores, EMA suicidal ideation ratings, and physical activity duration.

**Table S1. Missing-data handling and analytic sample summary**

| **Item** | **Value** |
| --- | --- |
| Scheduled day-level records | 2912 |
| Completed day-level records | 2358 |
| Incomplete day-level records | 554 |
| Completed records with non-missing evening SI | 2131 |
| Completed records excluded because evening SI was missing | 227 |
| Completed records excluded because lagged evening SI was missing | 0 |
| Day-level observations included in primary models | 2131 |
| Participants retained in analytic sample | 208 |
| Proportion of zero values for evening SI in analytic sample | 280/2131 (13.1%) |

*Note.* Missing EMA observations were not imputed. Primary models used all available completed day-level records with non-missing evening suicidal ideation; models including lagged evening suicidal ideation additionally required non-missing lagged values.

**Table S2. Random-effects comparison for the primary model**

| **Model structure** | **AIC** | **BIC** | **Log likelihood** | **Decision** |
| --- | --- | --- | --- | --- |
| Random intercept only | 13651.306 | 13707.950 | -6815.653 | Retained in the manuscript |
| Random intercept + random slope for within-person daily PA | 13648.795 | 13716.767 | -6812.397 | Tested; not retained for parsimony |

*Note.* A random slope for the within-person daily physical activity effect was tested in the primary model. It produced a modest improvement in AIC (ΔAIC = 2.511) but a higher BIC (ΔBIC = 8.817) and left the fixed-effect estimate for within-person daily physical activity essentially unchanged (random intercept only: β = -1.780; random slope: β = -1.808). The random-intercept-only specification was therefore retained for parsimony and consistency across models.

**Table S3. Standardized effect-size indices for the reported mixed-effects models**

| **Model** | **Marginal R²** | **Conditional R²** | **Pseudo R² (within)** | **Pseudo R² (between)** |
| --- | --- | --- | --- | --- |
| Model 1. Overall daily PA | 0.365 | 0.594 | 0.067 | 0.731 |
| Model 2. Individual and team-based PA | 0.370 | 0.595 | 0.068 | 0.736 |
| Model 3. PA × childhood maltreatment | 0.416 | 0.609 | 0.069 | 0.764 |

Note. Semi-standardized coefficients for key effects were as follows: Model 1 within-person daily PA = -0.081; Model 2 within-person individual PA = -0.062 and within-person team-based PA = -0.071; Model 3 within-person daily PA = -0.080 and the daily PA × childhood maltreatment interaction = -0.024.

**Table S4. Comparison of retained and excluded EMA participants**

| **Variable** | **Retained participants, n = 208, Mean (SD)** | **Excluded participants, n = 37, Mean (SD)** | **t** | **p** | **Cohen’s d** |
| --- | --- | --- | --- | --- | --- |
| CTQ total score | 42.76 (9.62) | 42.14 (9.05) | 0.38 | 0.703 | 0.07 |
| Baseline BSI score | 8.56 (1.68) | 8.62 (1.44) | -0.23 | 0.816 | -0.04 |

**Figure S1. Residuals versus fitted values for the primary model**


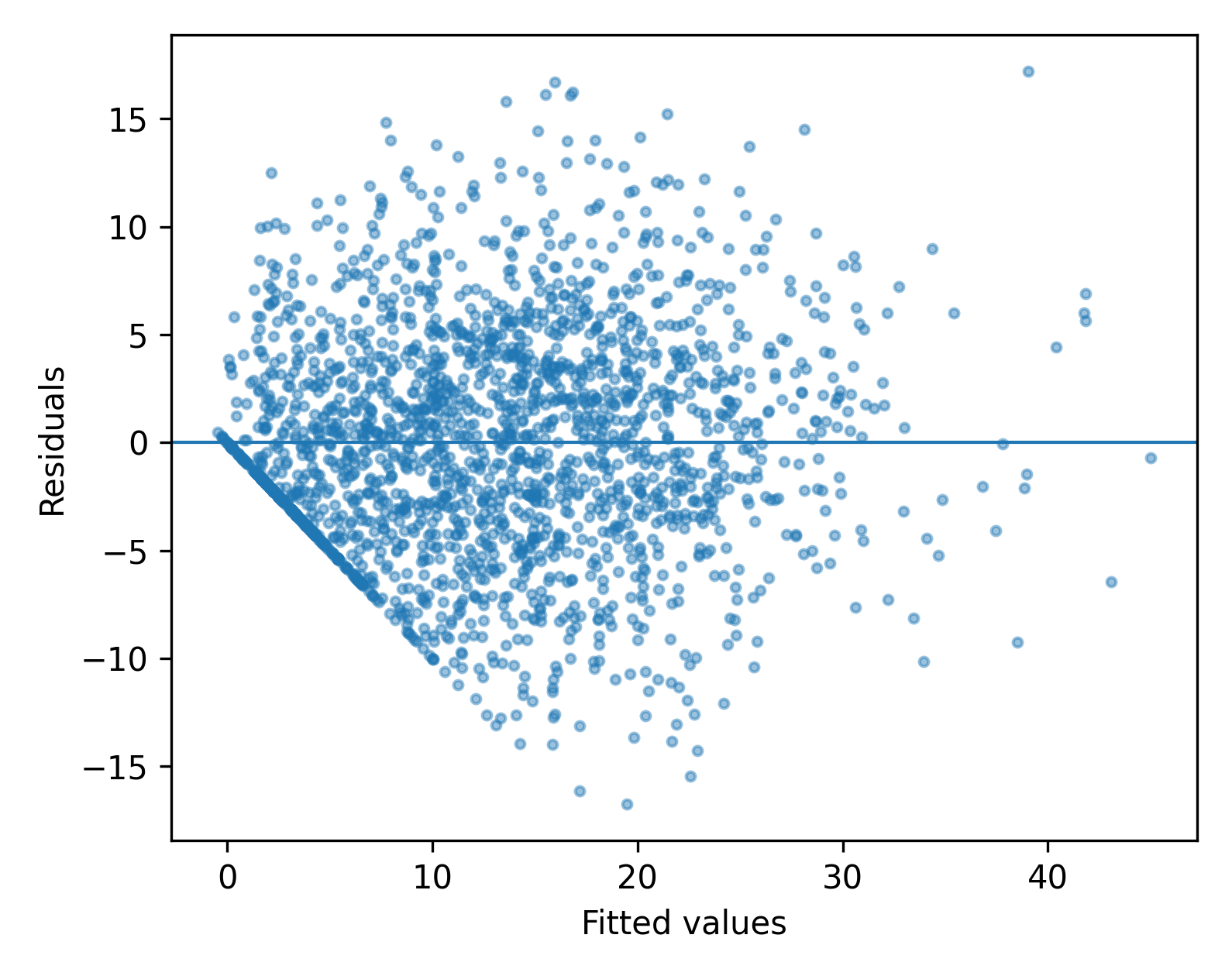


Note. Residuals were broadly centered around zero, although some heterogeneity in spread at lower fitted values cannot be ruled out

**Figure S2. Normal Q-Q plot of residuals for the primary model**


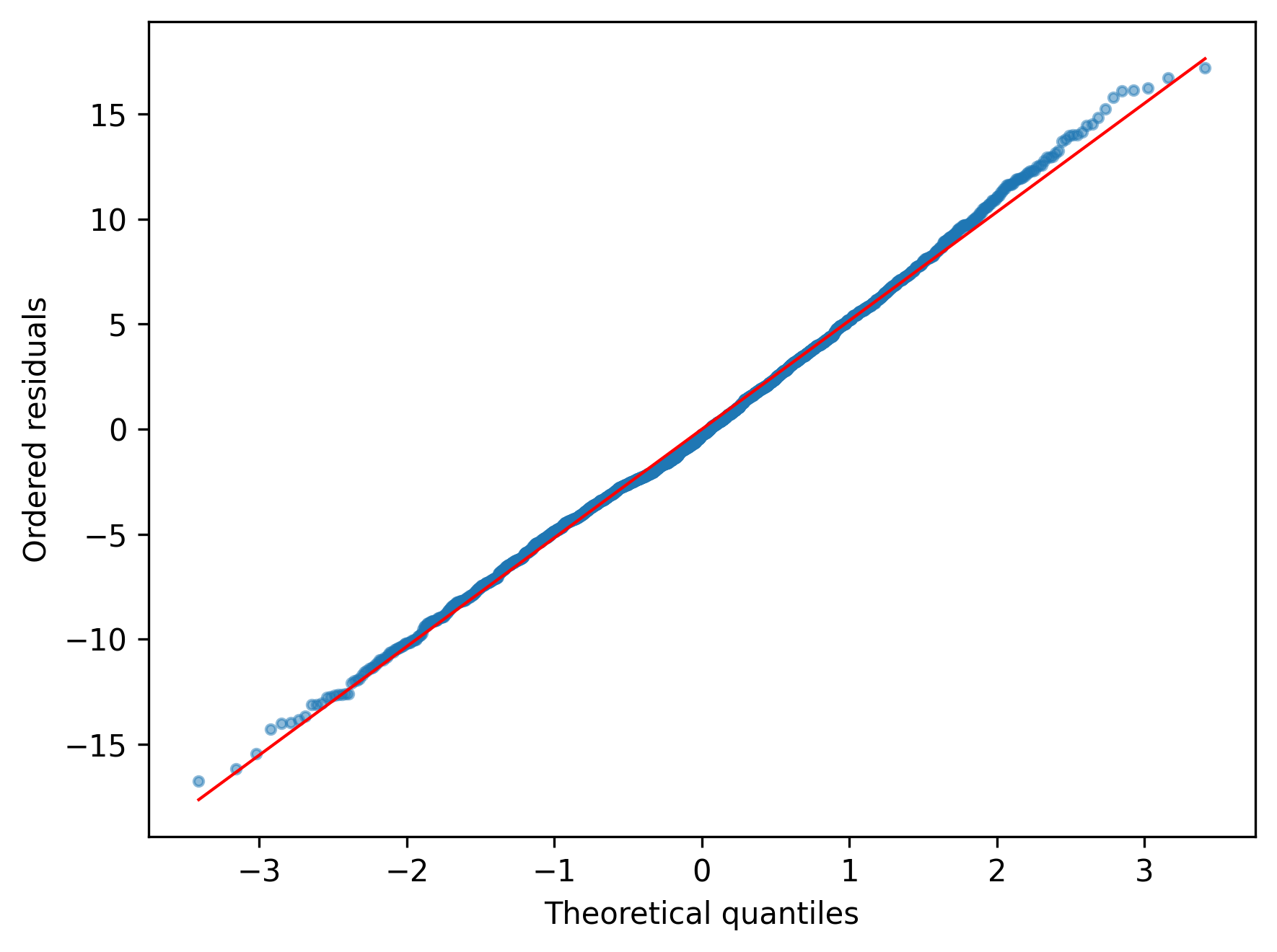


Note. The Q-Q plot suggested no substantial deviation from normality in the central portion of the residual distribution.

**Figure S3. Distribution of same-day evening suicidal ideation in the analytic sample**


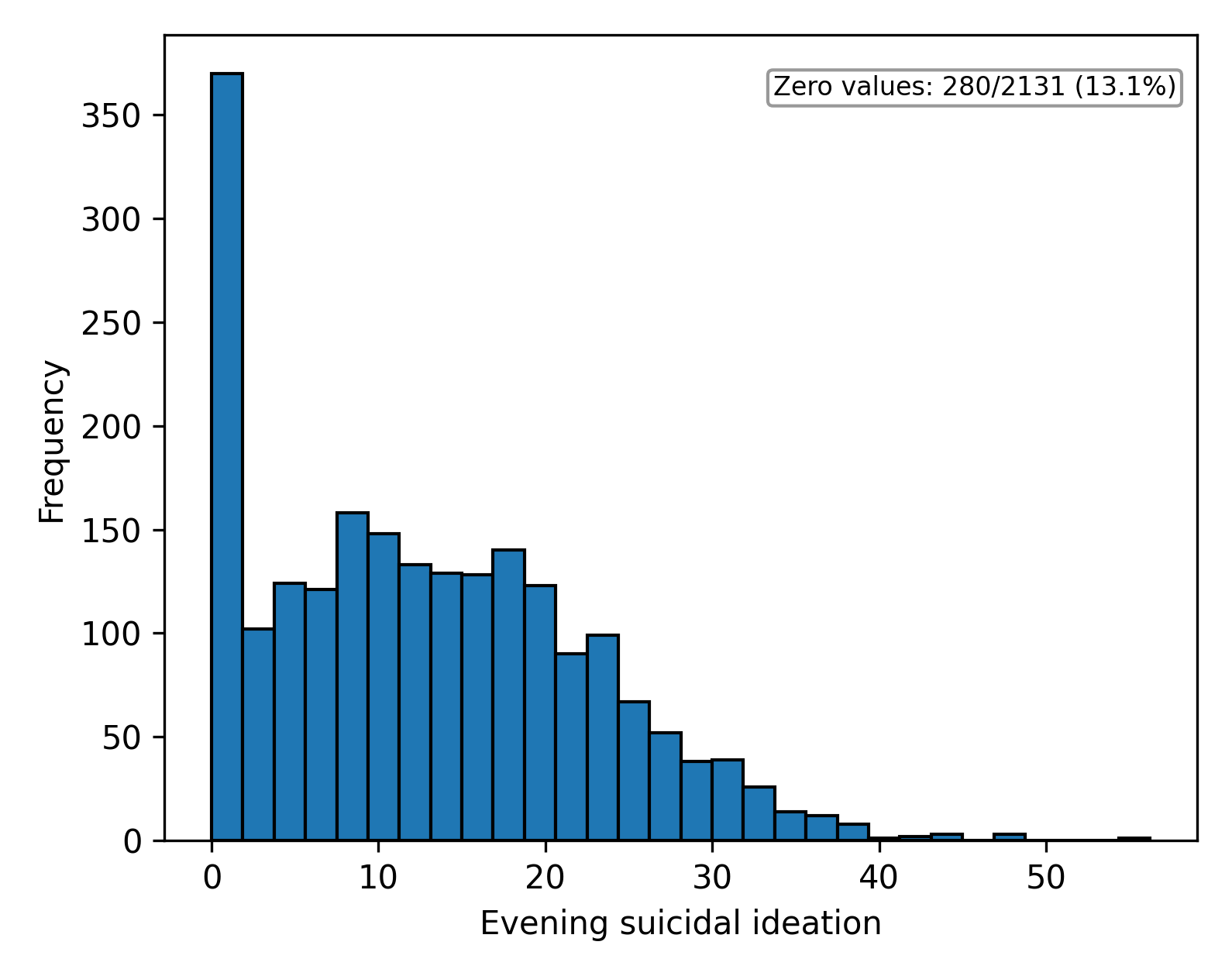


Note. Zero values accounted for 280 of 2131 analytic observations (13.1%).

**Figure S4. Distribution of baseline BSI total scores in the analytic sample**


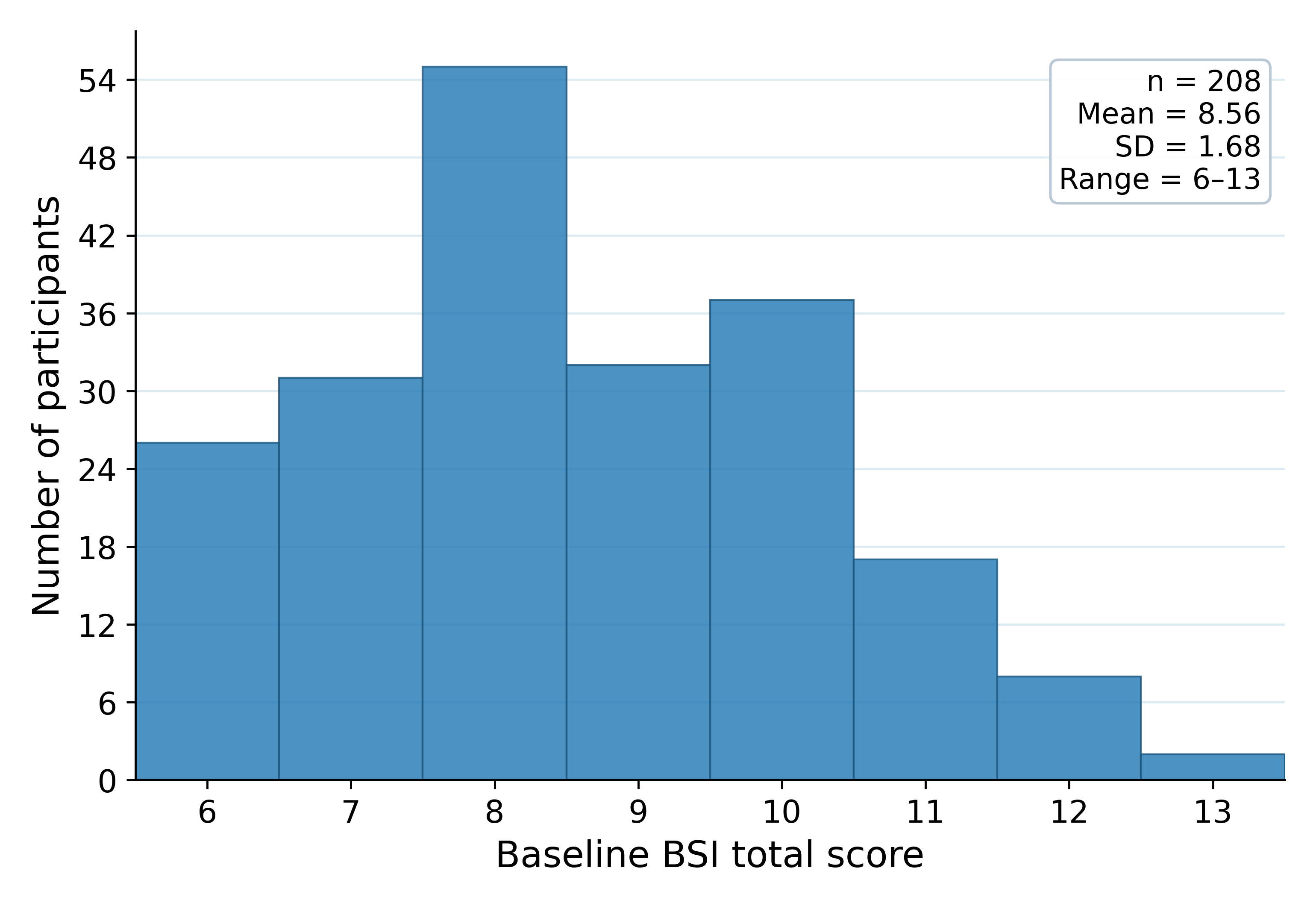


Note. Scores were restricted by the inclusion criterion of BSI >= 6; therefore, the lower bound at 6 reflects the screening design rather than the natural floor of the scale.

**Figure S5. Distribution of all non-missing EMA suicidal ideation ratings across morning, afternoon, and evening assessments**


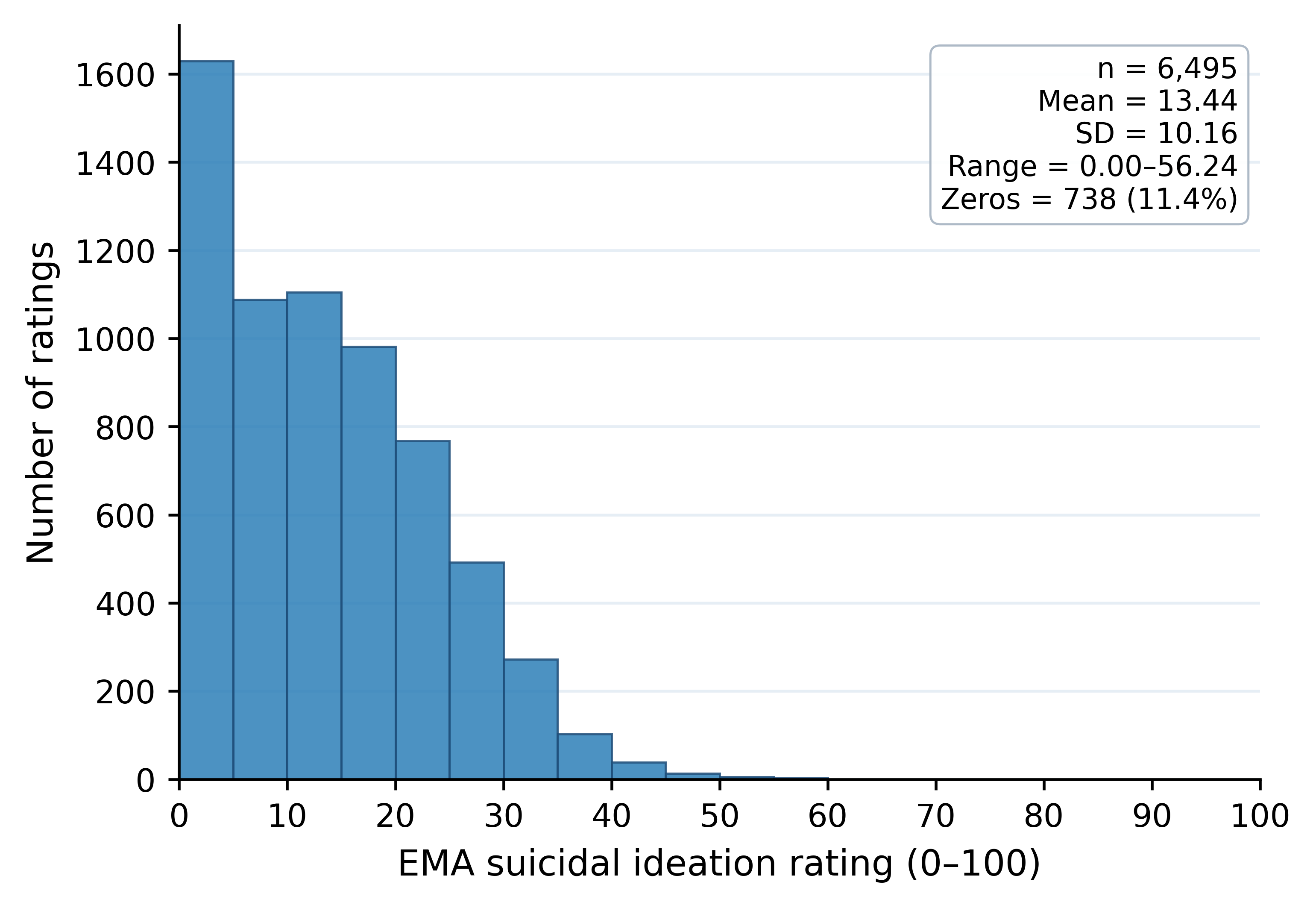


Note. Exact zero values accounted for 738 of 6,495 ratings (11.4%); no rating exceeded 56.24 on the 0-100 scale, indicating no ceiling effect.

**Figure S6. Distribution of physical activity duration on active completed days**


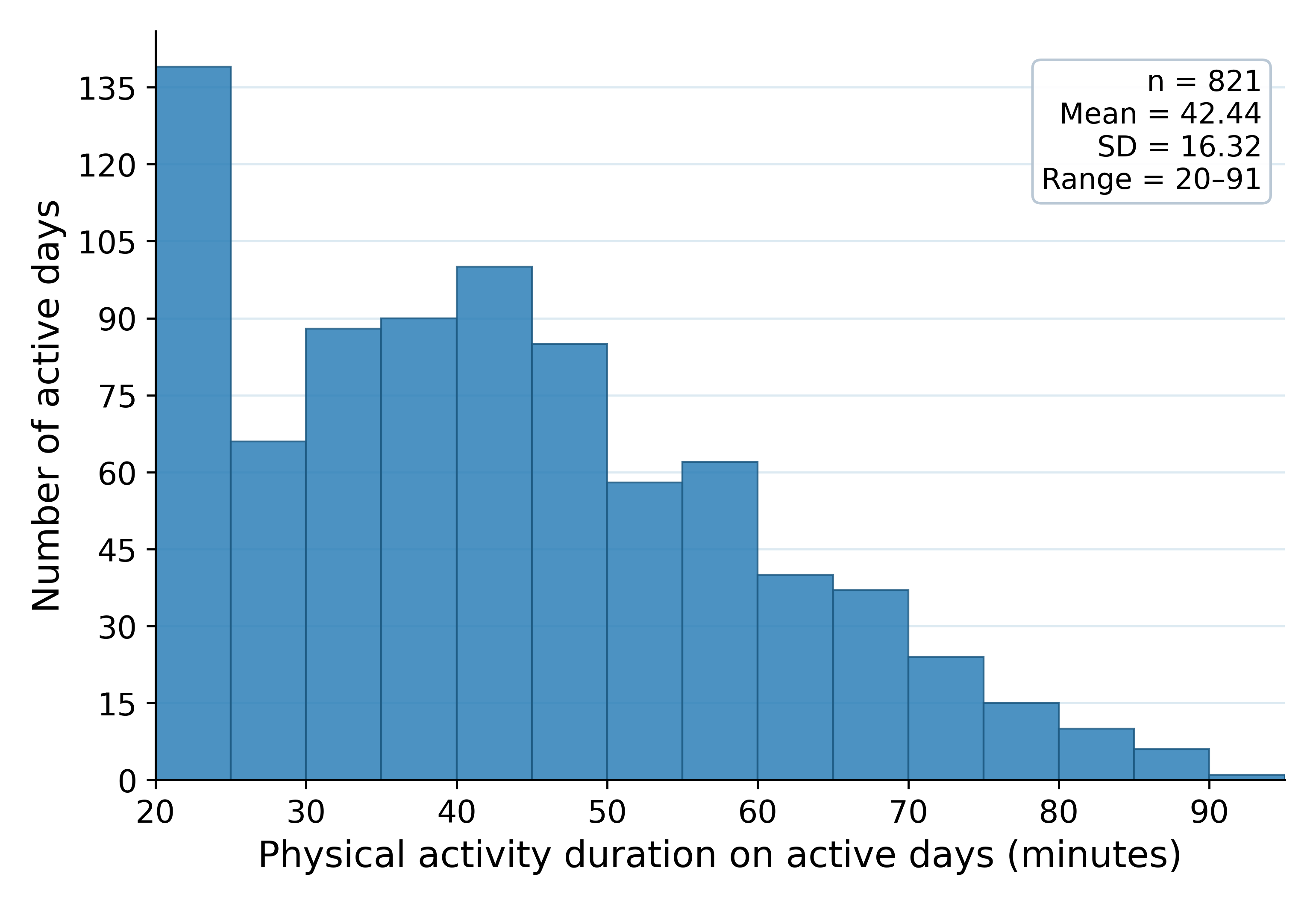


Note. Activity duration was summarized among active completed days only (n = 821).
